# A Survey of Genomic Traces Reveals a Common Sequencing Error, RNA Editing, and DNA Editing

**DOI:** 10.1371/journal.pgen.1000954

**Published:** 2010-05-20

**Authors:** Alexander Wait Zaranek, Erez Y. Levanon, Tomer Zecharia, Tom Clegg, George M. Church

**Affiliations:** 1 Department of Genetics, Harvard Medical School, Boston, Massachusetts, United States of America; 2 The Mina and Everard Goodman Faculty of Life Sciences, Bar-Ilan University, Ramat-Gan, Israel; 3 Compugen Ltd., Tel Aviv, Israel; 4 Scalable Computing Experts, Somerville, Massachusetts, United States of America; Friedrich Miescher Institute for Biomedical Research, Switzerland

## Abstract

While it is widely held that an organism's genomic information should remain constant, several protein families are known to modify it. Members of the AID/APOBEC protein family can deaminate DNA. Similarly, members of the ADAR family can deaminate RNA. Characterizing the scope of these events is challenging. Here we use large genomic data sets, such as the two billion sequences in the NCBI Trace Archive, to look for clusters of mismatches of the same type, which are a hallmark of editing events caused by APOBEC3 and ADAR. We align 603,249,815 traces from the NCBI trace archive to their reference genomes. In clusters of mismatches of increasing size, at least one systematic sequencing error dominates the results (G-to-A). It is still present in mismatches with 99% accuracy and only vanishes in mismatches at 99.99% accuracy or higher. The error appears to have entered into about 1% of the HapMap, possibly affecting other users that rely on this resource. Further investigation, using stringent quality thresholds, uncovers thousands of mismatch clusters with no apparent defects in their chromatograms. These traces provide the first reported candidates of endogenous DNA editing in human, further elucidating RNA editing in human and mouse and also revealing, for the first time, extensive RNA editing in *Xenopus tropicali*s. We show that the NCBI Trace Archive provides a valuable resource for the investigation of the phenomena of DNA and RNA editing, as well as setting the stage for a comprehensive mapping of editing events in large-scale genomic datasets.

## Author Summary

Most biomedical, genomic research begins with the painstaking assembly of a “reference genome” for the organism of interest. Implicit in this process is an assumption that genomic information is constant throughout an organism. There are enzymes, however, that can change, or “edit,” genomic information so that variations from the reference can exist within a single organism. In this work, we analyze the raw data used to assemble the reference genomes of ten organisms to discover evidence for editing. We found candidates for DNA and RNA editing as well as a sequencing error that has become incorporated into commonly used genomic resources. Our analysis demonstrates the utility of raw genomic data for the discovery of some editing events and sets the stage for further analysis as sequencing costs continue to decrease exponentially.

## Introduction

With the exception of infrequent random somatic mutations, it is widely believed that the same genomic content should be fixed in an organism throughout its lifetime. This information will also serve as a template for exact RNA copies. Proteins that can modify genomic content, nevertheless, have been identified in humans and in many other organisms.

RNA editing involves alteration of particular RNA nucleotides by specifically changing Adenosine (A) into Inosine (I), which in turn is read as Guanosine (G) [1]. It is performed by the adenosine deaminase that acts on RNA (ADAR) family of deaminases [2]–[5] and this process has been implicated in several vital neurological functions [6]. A-to-I editing is known to target only RNA molecules [7] with numerous instances of editing events in the human transcriptome [8]–[12]. A different family of proteins, the AID/APOBEC family of deaminases, can edit both DNA and RNA nucleotides, specifically changing Cytosine (C) into Uracil (U) [13]. The first family member to be found and studied was the apolipoprotein B editing complex 1 (APOBEC1). This protein edits the apolipoprotein B (ApoB) RNA, which is involved in lipid transport [14], [15] but APOBEC1 can also deaminate cytidine in DNA [16]. Additional members of the family were found to target DNA. Activation-induced deaminase (AID) was discovered to be vital for the antigen-driven diversification of immunoglobulin genes in the vertebrate adaptive immune system [17]–[19] and the APOBEC3s were shown to be involved in the restriction of retrovirus proliferation in primates [20], [21].

For many years, the only known human endogenous target of the APOBEC protein family was the apoB RNA transcript. In this case, editing in position 6,666 by APOBEC1 leads to a stop codon and eventually results in two functionally distinct isoforms of apolipoprotein B (ApoB) [15], [22]. This editing reaction is mediated by the APOBEC complementation factor (ACF) [23], [24] which guides APOBEC1 to the target locus.

Deamination of cytosines to uracils in DNA (DNA editing) by various APOBEC protein families is characterized, in many cases, by clusters of G-to-A mismatches between the reference genome and the edited sequence. These mismatches are the end product of deamination of “C” into “U” in the other DNA strand. Recently, it was found that APOBEC3G can serve as a potent inhibitor of a wide range of retroviruses, including endogenous retrotransposons. This protein introduces large numbers of C-to-U mutations in the minus-strand of the viral DNA, eventually leading to G-to-A mutations after plus-strand synthesis [25]–[29]. Also, it has been demonstrated that APOBEC3G is capable of editing the mouse IAP retrotransposon [30]. Little is known, however, about the frequency or localization of editing *in vivo*.

Although editing of retrotransposons and their integration back into the genome is expected to be rare, very deep DNA sequencing can be used to identify these events. In this paper we report initial results of a novel bioinformatic approach for detection of endogenous RNA and DNA candidate sites in various organisms. We obtained 600 million sequence traces from the NCBI Trace archive. This data repository contains DNA sequence chromatograms (traces) from various large-scale capillary electrophoresis sequencing projects, base calls, and quality estimates. Next, we aligned these traces to their consensus reference genomes and searched for clusters of mismatches. Interestingly, we have found not only evidence of genuine RNA and DNA editing events but have also isolated a very common technical sequencing artifact that leads to such clusters.

## Results

One hallmark of editing enzymes is a cluster of mismatches of the same type in the edited substrate. While the results of the RNA editing ADARs are clusters of A-to-G mismatches, the hallmark of members of the APOBEC3s protein family is a cluster of G-to-A mismatches in the newly formed DNA strand after reverse transcription. In order to find new endogenous editing events we looked for such mismatch clusters in the largest available repository of “raw” sequencing data, before they have been processed and assembled. We aligned “raw” sequencing reads from the NCBI trace archive to their consensus, reference genome. We repeated this procedure, in parallel, for each of ten organisms (in total more than 600 million reads - see Materials and Methods). In order to reduce noise caused by low sequencing quality or from misalignment to the genome, only long alignments (400bp or more) with 97% identity to the reference were considered. In addition, no insertions or deletions and no ambiguity in the location of the alignment were accepted (see Materials and Methods). Applying these strict criteria we do not expect results from current ABI SOLiD, Roche 454, Illumina GA, or Helicos sequencing reads.

In sum, we curated more than 56 gigabases of aligned sequence in human, about 62 gigabases of aligned sequence in mouse and much lower numbers for other organisms reflecting smaller genomes and/or lower coverage. In human, 85,181,171 traces aligned uniquely to the reference genome, 4,626,984 traces aligned to multiple locations, and 123,110,314 traces had no alignment under our strict cutoffs. For all organisms combined, approximately 300 million, out of 603,249,815 traces in total, were analyzed further (See Table 1).

**Table 1:** Summary of computation.

Organism name	#reference bp (millions)	#unique traces (millions)	Mean coverage	Space (Gb)	Time (millions of node seconds)
*Anopheles gambiae*	260	4.3	9.9	13	0.56
*Callithrix jacchus*	2,900	22	4.6	160	1.5
*Canis familiaris*	2,400	33	8.3	370	3.4
*Drosophila melanogaster*	160	0.67	2.5	2.5	0.06
*Gallus gallus*	1,000	12	7.2	30	1.3
*Homo sapiens*	2,900	85	18	530	30
*Mus musculus*	2,600	93	21	4,200	114
*Pan troglodytes*	2,900	32	6.6	150	7.0
*Takifugu rubripes*	350	2.5	4.2	6.4	1.2
*Xenopus tropicalis*	1400	14	6.0	360	4.8
**Total**		298.47		5821.90	163.82

Total data generated from analysis of 603,249,815 traces, 30% of the total number of traces at NCBI (outside the short-read archive). Approximately half were placed uniquely while applying our cutoffs, with total data consuming six terabytes of disk and more than five “node years”of CPU time. The computation on mouse traces produced the bulk of the data.

Clusters of consecutive mismatches of the same type (C-to-T or G-to-A) are common in APOBEC targets, such as IAP mouse retroelements edited by APOBEC3 [30], thus we focused on such “runs” in the aligned traces. In human, we found G-to-A mismatches to be over-represented compared to other types of mismatch, with longer runs. There were 657,826 human traces with runs of five or more mismatches of the same type. Of these, 218,595 (33%) human traces had runs of five or more G-to-A mismatches, much more than any other mismatch type.

Since editing enzymes have a preferred sequence context, the large data set allows us to restrict our search to traces with the same three base-pair motif centered at each mismatch site in the trace [31]. Moreover, as sequencing errors tend to cluster in certain regions, especially in low complexity areas, hence forming relatively short mismatch-dense regions, we applied another filter and discarded runs that span less than 100 base-pairs (the distance between the first and last consecutive mismatch). We also discarded traces in which the reference or the trace nucleotides around or at the mismatch site were not called (“N”).

**Figure 1:**
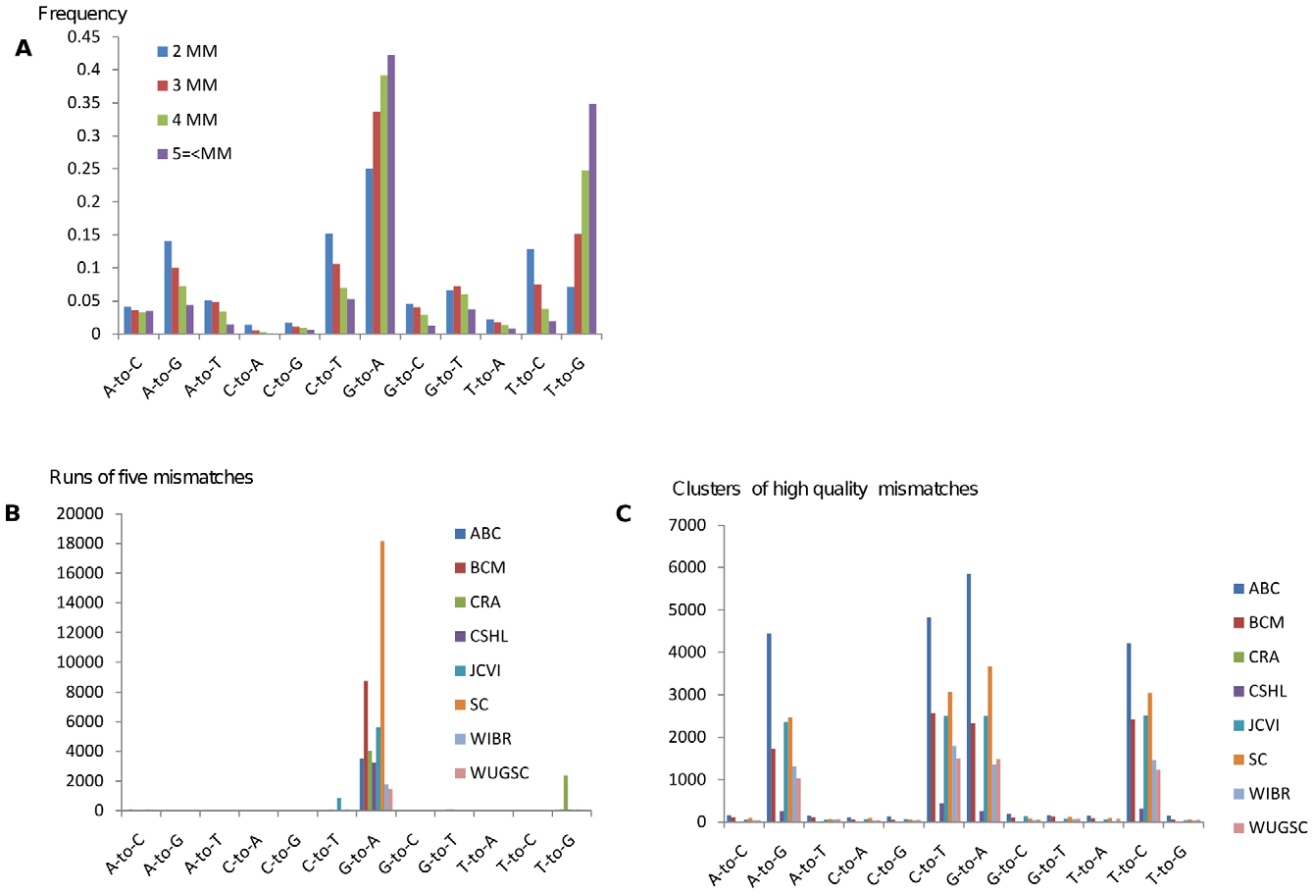
Evidence for editing events emerges by enrichment for clusters of mismatches. (A) Human traces are mined for clusters of mismatches of the same type. Shown is the percent frequency of clusters by type. The G-to-A mismatch type becomes more dominant with increasing numbers of mismatches (as does T-to-G). (B) Runs of five (or more) mismatches by type and sequencing center with an identical 3bp motif centered on each mismatch. Data from eight sequencing centers is shown. All of these centers had at least 1000 examples that meet the above criteria. (C) Clusters with three (or more) mismatches with at least two very high quality mismatches (Phred 40). A mismatch spectrum consistent with editing can be observed.

Out of the 53,639 total examples conforming to the above criteria, we found 46,483 (82%) examples of G-to-A traces in human. Thus, the restrictions above reduced the total number of traces more than 12-fold while only reducing the number of G-to-A examples by less than 5-fold. Moreover, we found a striking preference for either an “AGA-to-AAA” mismatch motif (26,694/53,639 traces) or an “AGG-to-AAG” motif (21,274/53,639 traces). This tendency was observed in traces from all sequencing centers tested but one (Celera) (see Figure 1B). Since most of the sequencing for the human genome project was done in eight centers, results from only these centers are shown.

Sequence traces are derived from both DNA strands, thus one would expect to observe a symmetric over representation of C-to-T mismatch clusters. Lack of similar numbers of complementary mismatches led us to the conclusion that most of these mismatches are not caused by a biological source but rather are sequencing artifacts.

**Figure 2:**
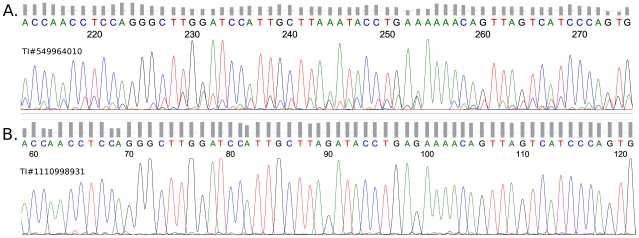
G-to-A sequencing artifact. (A) A chromatogram, from a trace matching the criteria in Figure 1B. An AAA motif is centered at position 244 and corresponds with position 90 in the control; another AAA motif occurs at position 253 which corresponds to position 99 in the control. It can be seen that each peak in this chromatogram is preceded by a smaller, identical sub-peak. This has the effect of making it likely that a normally small peak (see control) will be overwhelmed by the sub-peak of the adjacent, normally tall peak (see control). (B) A chromatogram from a control trace that matches the reference—position 90 is the center of an AGA motif.

In order to understand the origin of the artifact, we analyzed sample traces, and noticed that traces with “runs” of mismatches, with identical three base-pair motifs, centered on the mismatch, often had a peculiar defect in their chromatograms. Such defects can arise when the florescent dyes used in DNA sequencing have sequence specific incorporation differences which lead to unevenly spaced or shaped peaks in the electronic trace chromatogram after capillary electrophoresis. Figure 2 shows a comparison of representative chromatograms: one with the “AGA-to-AAA” motif (Figure 2A) and one that matches the consensus genome (Figure 2B). A mismatch is highlighted at position 244 and matches position 90 in the control. We can see that every peak is preceded by a small, identical sub-peak. There is also another “AAA” motif at position 253 which corresponds to an “AGA” motif at position 99 in the control. Independently, we noticed that “AGA” sequences are prone to form a pattern of high, low, high intensity peaks, hence the “G” has a low peak while the preceding and the subsequent “A” peaks are much taller (see control). The combination of these two common effects, in one trace, leads to occurrences where the sub-peak from the high “A” can dominate the “G” resulting in a G-to-A mismatch in an “AGA” context.

We used strict criteria to construct the artifact set, thus the actual number of those errors is probably much larger than the 260K we found and may disrupt the accuracy of genomic assemblies. Indeed, we found evidence that these common errors influence the consensus sequence of a few genomes. The number of runs of G-to-A mismatches with the AGA motif was much higher in genomes with high coverage, where each position in the reference genome has many traces to support each call. In these cases, the reference is determined according to the “majority voting” of all the supporting traces. Since the reported type of mismatch is much less abundant than the correct call, the reference will have the correct “G” in virtually all cases. In genomic projects with lower coverage, however, such events can become part of the reference genome and therefore could not have been detected by our method. Indeed, we found that genomes with lower coverage tended to be free of G-to-A mismatches. This effect is most striking in drosophila where mean coverage of the reference by aligned traces is only 2.5 (See Table 1 and Table 2). This finding suggests the integration of these sequencing errors into the reference genome in many cases.

**Table 2:** Editing enriched traces—higher quality.

Reference genome version	G-to-A	C-to-T	A-to-G	T-to-C	Other
anoGam1	2836	2830	2907	3098	440
calJac1	3012	3362	2735	3133	145
canFam2	3170	3777	3270	3027	212
dm3	1	1	0	1	0
galGal3	1290	878	1026	1760	48
hg18	17719(82)	16778(72)	13701(188)	15301(419)	700(8)
mm9	1801(219)	1644(272)	1346(276)	1411(346)	76(11)
panTro2	3485	3120	2918	4046	240
fr2	467	449	390	482	45
xenTro2	1483(202)	1574(262)	1461(1289)	1631(1066)	269(28)

Number of traces by mismatch type with two or more mismatches at or above a quality threshold of phred 40, spanning 100bp or more. All mismatches belong to runs of three consecutive mismatches of the same type of any quality. The number of traces from the next largest substitution type, or the largest substitution type if it is not one of A-to-G, T-to-C, G-to-A, or C-to-T, is shown in the “other” column for comparison. The numbers in parentheses indicate traces of RNA origin. See Materials and Methods for more details.

Another effect of this error was found in the assignment of single nucleotide polymorphisms (SNPs). A sequencing error in one genomic trace will not usually lead to the determination of a SNP at this position. However, since many of the “AGA” mismatches have a quality score of phred 20 or higher, which is considered an acceptable quality with an estimated error probability of only 1% [32] we suspected that some of them might be classified as SNPs. Indeed, we found 46,483 traces with 3bp G-to-A motif in runs of five or more. Of ∼260K G-to-A mismatches with this motif, we found that 28,722 appear as SNPs in dbSNP (The Single Nucleotide Polymorphism database) and 11,145 even appear in the HapMap dataset and were genotyped in four populations. Strong support that the vast majority are actually errors and not real SNPs comes from the observation that 10,532 (94.5%) of the mismatches that appear in HapMap are homozygous for the reference allele (G) with no representation of the other SNP allele in any of the 90 individuals that were genotyped in the Yoruba population, a population that is typically the most diverse. By contrast, only 521,405 out of the 3,782,819 (13.8%) of SNPs that appear in the HapMap show a similar lack of variability (*p*-value≪e-200, Fisher's Exact Test). Over-representation of G as the observed allele in A/G SNPs (or C in C/T SNPs) in the group of SNPs that have only one observed allele, when comparing it to the SNPs with two observed alleles, suggests that up to 1.8% of the HapMap SNPs are a result of the artifact and are not real SNPs (data not shown) the ratio is probably larger in dbSNP which is less curated.

Once we realized that the majority of “AGA” and “AGG” mismatch motifs were caused by a sequencing error, we endeavored to eliminate such errors from our dataset. To do so, we incorporated phred quality scores, also available from the trace archive. We obtained quality scores for all traces with a run of three or more substitutions of the same type. This set contains 20.7 million traces out of the 300 million that aligned uniquely. We then applied various quality score thresholds on to the data (see Materials and Methods). At quality scores above phred 40, where the chances of incorrect calls are just 1 in 10,000 [33], the number of G-to-A substitutions becomes roughly equivalent to C-to-T, A-to-G and T-to-C substitutions, in agreement with the current knowledge of mutations and the expected distribution of SNPs. This suggests that the systematic sequencing error we detected is diminished at such high phred values and traces are further enriched for genuine editing sites (Figure 1C).

### DNA editing

Recently, DNA editing has been reported to be a powerful defense mechanism against the threat of genomic instability imposed by viruses and retrotransposons. However, the full magnitude of the phenomenon *in vivo* is not yet elucidated. We wanted to investigate whether our curated dataset of G-to-A mismatch clusters may actually include some examples of DNA editing. To test this assumption we looked at mismatch clusters in the mouse genome. We found that the total number of A-to-G and T-to-C mismatches was similar to the number of C-to-T and G-to-A mismatches (7,860 vs. 9,799). However, in genomic regions of *IAP* (intracisternal A-particle) elements, for which a few members are still active, there was a significant dominance of the G-to-A / T-to-C mismatches (114 compared to 49 A-to-G / T-to-C) (*p*-value of 0.00018, Fisher's Exact Test). This supports the idea that the origin of the mismatches is a result of editing by APOBEC after reverse transcription of the retrotransposons. An example of a DNA editing candidate, in a mouse retrotransposon, is given in Figure S1.

**Figure 3:**
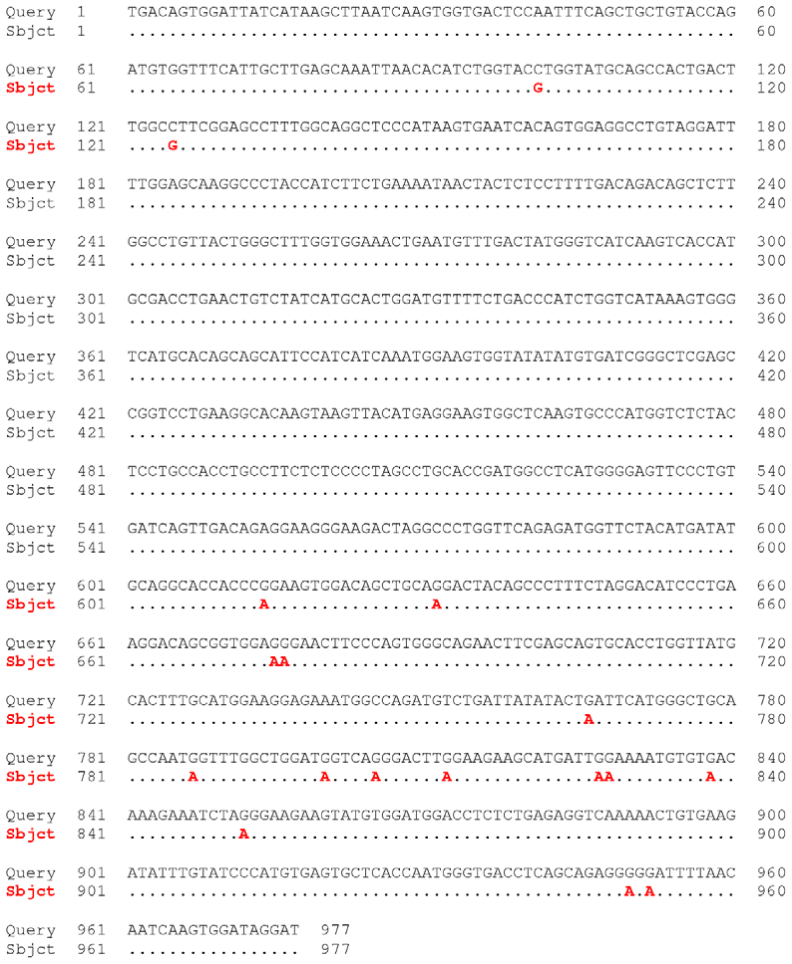
DNA editing in human HERVL-A1. Trace 1735626615 aligns uniquely to chromosome 2 where the known retrotransposon HERVL-A1 is located (chr2: 100697697–100700125). A cluster of 15 G-to-A mismatches (worst mismatch phred 35; best mismatch phred 49) suggests that the trace originates from an edited version of the element. Support for the APOBEC source of the editing comes from the preferred GG-to-AG motif (11 out of the 15 cases) and GA-to-AA (remaining 4 cases) which is the dinucleotide context (in the same order) in an HIV hypermutated genome, and is the sequence motif of APOBEC3G and APOBEC3F [31].

**Figure 4:**
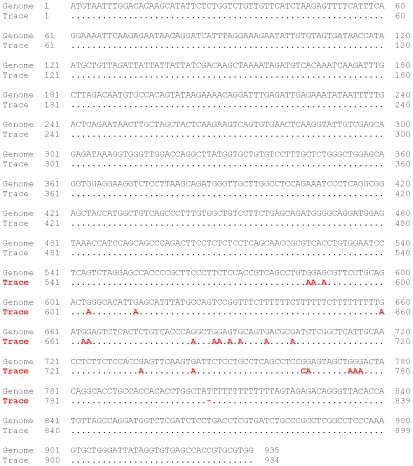
DNA editing in human AluY. Example of possible DNA editing in human chr21:40977741–40978045. Alignment of trace 1745107496 to the human reference genome lead to large number of G-to-A mismatches which are indications for possible DNA editing in this retrotransposon. All the mismatches are located in high quality sequence positions, reducing the possibility of sequence errors.

Active retrotransposons exist in human. For example, two edited HERVK elements have been recently discovered [34]. Thus, we applied our approach to human genomic sequences. Indeed we found evidence for DNA editing. We detected 247 events of G-to-A / C-to-T mismatch clusters versus 129 A-to-G / T-to-C events (while overall in the genome the ratio is 91,120 to 79,401 respectively) (*p*-value of 0.0000017, Fisher's Exact Test). One such candidate of editing by APOBEC in human retrotransposon HERVL-A1 is shown in Figure 3. An additional example for a probable editing event in a human retrotransposon is present in Figure 4 where clusters of G-to-A mismatches are found in the most active SINE family in human, *AluY*. All of these mismatches have high sequencing quality (Phred 40 or greater). Moreover, previously it was demonstrated that APOBEC3 can inhibit retrotransposition of Alu [35].

The actual number of edited traces in the trace archive is most probably much higher than we have found, for several reasons: More than half of all traces were rejected with our alignment parameters, at least partially due to the fact that DNA editing tends to lead to hyper-mutation in its target sequences [31]. Furthermore, we expect that a significant number of traces from retrotransposons, which are known targets for the APOBEC in their cDNA stage, are too redundant to align uniquely. Indeed, we found that in many cases the second best alignment of a putatively edited trace almost qualified for the 97% cut-off criteria, meaning that the trace was close to being rejected for having multiple possible genomic alignments. Thus, future work should find ways to curate the data in a less stringent manner so that editing, in traces with multiple hits to the genome or that do not meet our identity cut-offs, can still be detected. This would foster the development of a more complete picture of the occurrence of DNA editing in mammalian genomes.

### RNA editing

RNA editing is a general term for the modification of RNA after it is transcribed from DNA. The most common modification in mammals is A-to-I editing by the ADAR protein family. As I (Inosine) is read as a G (Guanosine) after sequencing, this editing type manifests itself as an A-to-G substitutions after cDNA sequencing and alignment to the original genomic locus. Recently it was found that the human genome harbors large numbers of editing events that are located in clusters, mainly in *Alu* repeats [9], [10], [11], [12]. The origin of mismatch clusters in some of our traces, therefore, can be the result of ADAR activity.

**Figure 5:**
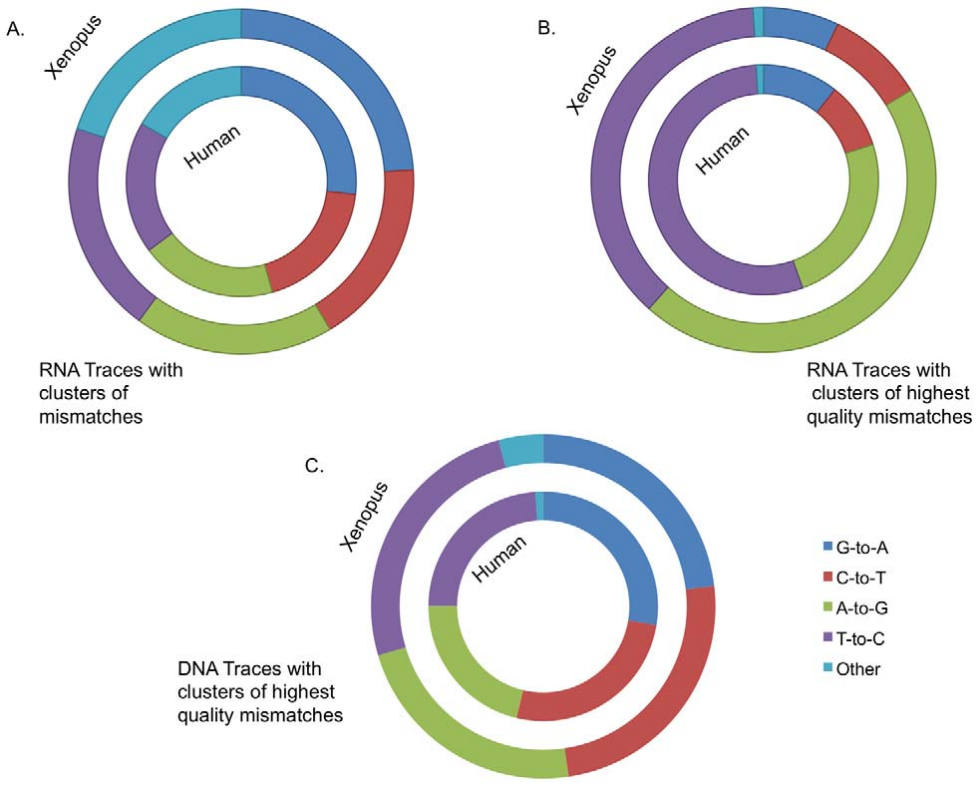
Evidence for RNA editing in the cDNA traces. (A) While no over-representation of the RNA derived mismatches (A-to-G and its complimentary T-to-C) clusters are observed in the full set of RNA traces in human (n = 238,370) and *Xenopus tropicalis* (n = 444,526), (B) significant over-representation of RNA editing type is observed in high quality cDNA sequencing set of human (n = 769; *p*-value 1.5e-119; Fisher's Exact Test.) and Xenopus (n = 2,847; *p*-value≪e-200). (C) No such over-representation was observed in the set of high quality DNA traces (human: n = 64,191; Xenopus: n = 3,471). These observations support that RNA editing is the cause of the mismatches in the sets of higher quality cDNA.

A fraction of the human, mouse and *Xenopus tropicalis* sequences obtained from the trace archive are labeled as derived from RNA, rather than DNA. In total, after passing the stringent alignment criteria, 250K, 513K and 454K traces, respectively, of those genomes have RNA origin, thus A-to-G or T-to-C mismatches in these traces could be the result of RNA editing. No over-representation (38% of the total MM clusters) of A-to-G or T-to-C clusters appear in the RNA trace set (Figure 5A), but as demonstrated above, the vast majority of mismatches are probably derived from a sequencing artifact. To overcome this issue we filtered those RNA traces and generated a higher quality, enriched set which required 3 consecutive mismatches of any quality and two mismatches separated by at least 100bp of phred 40 or greater. When we consider our higher quality, editing enriched set (See Figure 5B), we find, in human, over-representation of mismatches that can be the result of RNA editing (A-to-G and T-to-C), a total of 79% of the mismatch clusters are now of this type (*p*-value 1.5e-119; Fisher's Exact Test.) These observations suggest that RNA editing is the cause of the mismatches in the higher quality RNA sets.

**Figure 6:**
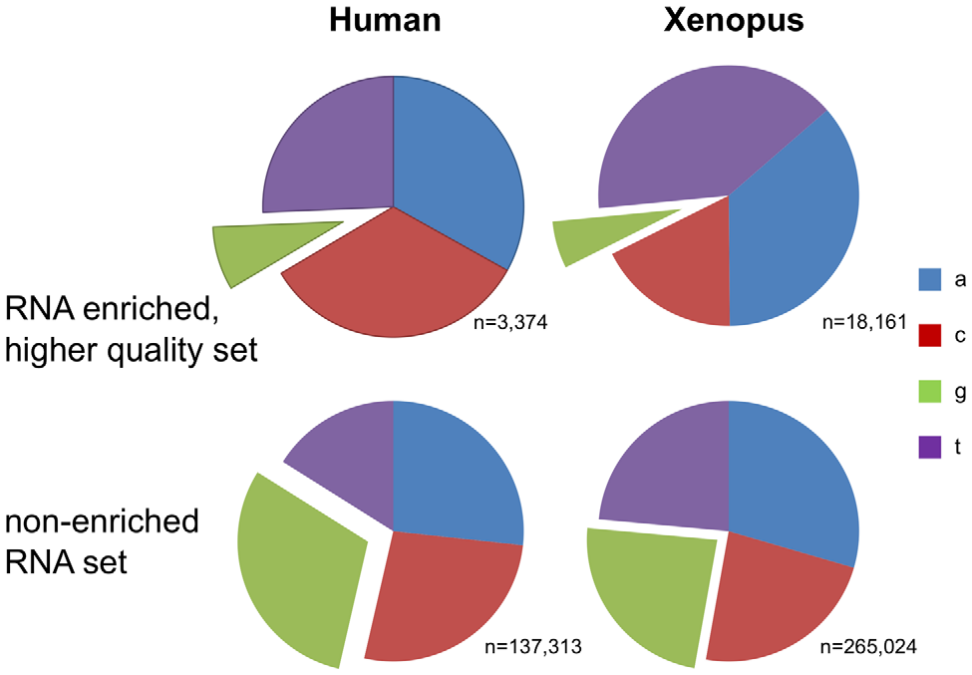
ADAR signature in the cDNA edited traces. Significant under-representation of “G” immediately upstream to the editing sites which is in agreement with the known sequence motif of the ADAR proteins.

Further evidence that the higher quality set is indeed a result of RNA editing comes from two additional observations. First, a significant under-representation of “G” immediately upstream to the editing sites which is in agreement with the known sequence motif of the ADAR proteins [36]. In the enriched, higher quality set there was a G upstream of the mismatch in only 7.85% (265 out of 3,374) of the cases versus 30.3% (41,661 out of 137,313) in the non-enriched set (*p*-value 1.9e-143) [36],[37](See Figure 6). Second, most known editing events in human are located in *Alu* repeats and indeed 72% of the mismatches in the higher quality set are located in *Alu* repeats while *Alu* represents only about 10% of human DNA (*p*-value of 1.7e-110).

Detection of RNA editing from short EST sequences has proven to be challenging, due to their relatively low sequence quality [38] and indeed, almost all A-to-I sites found until now were detected from alignment of a small set (<200,000) of full length RNAs [9], [10], [11], [39]. In the present work we used the human EST data deposited in the trace archive (currently including 2M ESTs which are mostly derived from poly-A mRNA) and found thousands of potential editing sites. Only 156 sites out of the 3374 sites in the higher quality, enriched set overlap with the known set of about 20,000 editing sites reported by alignment of RNA to the genome (total of 3,218 new sites). This suggests that ESTs, after accounting for sequence quality, can serve as a rich source for RNA editing site predictions.

Of the organisms we studied, only human, mouse and *Xenopus tropicalis* had significant numbers of RNA traces. If we use our enriched, higher quality set as a proxy for the total number of editing events, our data shows that in mouse, editing occurs at an estimated rate of 1 mismatch per 100,000 unique, expressed base-pairs. In human, in agreement with previous publications [11], [39], [40], our figures show ten-fold higher frequency. A striking picture emerges in *Xenopus tropicalis*. A closely related species, *Xenopus laevis*, is a principal model organism for the study of RNA editing as ADAR activity was first described in *Xenopus laevis* oocytes [41] and recently, research on hyper edited sequences in *Xenopus laevis* lead to the suggestion that editing can down-regulate gene expression *in trans* [42]. Only one endogenous hyper editing target is known in Xenopus - basic fibroblast growth factor (bFGF) [43], [44]. Using our approach for detection of RNA editing we have observed significant over-representation of A-to-I derived mismatch in *Xenopus* t*ropicali*s. In the enriched set 83% of the mismatch clusters are of the A-to-G and T-to-C type, while these types contribute only 39% of the mismatch clusters in the non-enriched set (*p*-value≪e-200) (Figure 5). This strongly suggests that the mismatches in the enriched set are caused by RNA editing.

**Figure 7:**
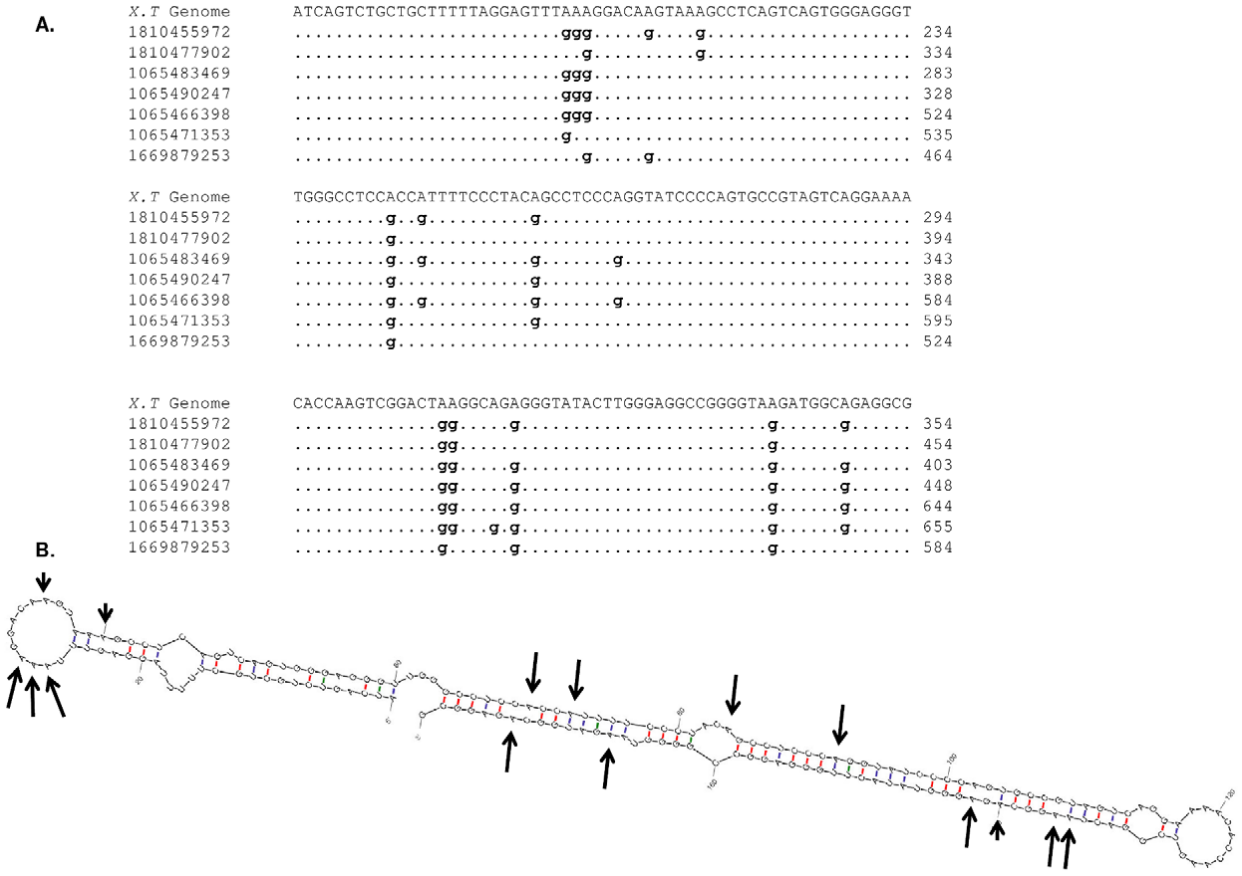
RNA editing in *Xenopus tropicalis*. (A) Evidence for RNA editing can be seen in this locus as multiple traces of RNA origin align to it with numerous A-to-G mismatches. The trace accession numbers and their coordinates are given in the multiple alignment. (B) Predicted RNA structure of the genomic locus indicates a long and stable dsRNA structure which is a favorite target for editing by ADARs. Each editing site from the multiple alignment is marked by an arrow. The length of the arrow corresponds to the editing level.

The *Xenopus tropicalis* genome has not been completed yet and the annotation is still partial. Thus, we cannot determine if the editing sites are located in one type or a small number of genomic repetitive regions. Interestingly, we found that 10001 out of the total 18161 mismatches in our editing-enriched, higher quality set occur in clusters of ten sites or more, larger than the common clusters detected in human RNAs sequences which have a typical size of less than 6 mismatches. By further examining a few mismatch clusters, we found that they tend to occur in palindromic regions that can form tight double stranded RNA. These structures are known to be required for ADAR editing (See Figure 7). As in human, we observed the ADAR signature of low abundance of “G” upstream of editing sites (5.8% for the higher quality enriched set versus 24% in the non-enriched set) (Figure 6, Tables S1, S2, S3, S4.). A full list with genomic coordinates of RNA editing sites in human and Xenopus is given in Datasets S1, S2.

## Discussion

The NCBI trace archive serves as a repository of raw data for the assembly of consensus genomes. Recently, it was utilized for a different purpose in the search for structural variation in the human genome [45]. Here, we show that it can also be used in the search for DNA and RNA editing. In the future, sequencing results deposited in the NCBI short-read archive might shed more light on these phenomena. Shorter reads, however, will pose a more challenging analysis problem.

Recently, we did an initial analysis of Illumina's human resequencing reads and the SOLiD reads from the same individual. These reads are available at the NCBI short-read archive and are the basis for the first individual African consensus genome [46], [47]. Given the importance of read-length and quality scores on the outcome of our current work, the current SOLiD and Illumina reads represent interesting trade-offs for the detection of editing. While Illumina's current read lengths are generally longer than SOLiD, the latter has much higher per-base quality. Adapting the techniques presented here to this new data presents an interesting opportunity for future research.

The availability of computational resources for carrying out our analyses was essential to this project, as large computational effort was needed, six terabytes of disk for intermediate data and more than five “node years” of CPU time. With further computational effort, combining existing data in the trace archive with next generation sequencing data sets from multiple sequencing platforms and chemistries, it should be possible to greatly improve genomic databases and eliminate the sequencing errors reported here.

By using well-calibrated quality scores and selecting traces with clusters of consecutive mismatches, we are able to investigate the scope of RNA editing sites in human and other genomes. The application of this technique in the search for editing events will make many large EST datasets more accessible for other organisms where quality scores are available. Currently, only a very small number of organisms, with large sets of full length RNA sequences, have been the subject of large-scale editing studies. Using quality scores, many additional genomes can be surveyed for editing with the opportunity for new discoveries in this emerging field.

As a demonstration of the value of using quality data for ESTs, we are able to find a large number of candidate RNA editing events in *Xenopus tropicalis*. This discovery makes *X. tropicalis* the non human organism with the largest number of known editing sites so far. Since Xenopus is already an important model organism for the research of RNA editing, this new data-set could help foster new discoveries in this field.

Despite the identification of thousands of newly discovered RNA editing sites in the current work, it is reasonable to believe that the actual number of editing sites is still significantly under-estimated. Support for this assertion comes from the stringency of our parameters: including length of alignment, percentage of identity and exclusion of insertions or deletions. These choices most likely limited the subset of EST data that we analyzed. Refinement of these criteria could lead to more comprehensive detection of RNA editing levels and, due to the breadth of EST data, even permit the comparison of editing levels in different tissues and disease conditions.

In this work we also found evidence for recent or active events of DNA editing. While the true scope of these phenomena must be explored in future work, our approach, including the use of strict alignment criteria and quality scores, has proved effective at finding many intriguing examples. Using different parameters, mainly lower cutoffs and relaxation of the requirement for unique alignments, more DNA editing sites could be detected in the trace archive. Careful investigation, most likely combined with next-generation sequencing experiments, will help unravel the mechanisms of retroelement defenses in a variety of organisms. Moreover, DNA editing is known not to be limited to retrotransposons and can take place in other genomic loci. The most recognized example is the AID protein, which is a member of the AID/APOBEC protein family, and targets single stranded DNA in the immunoglobulin locus in B-cells. Similar approaches to the ones used here provide an exciting opportunity to survey how leakage of DNA editing events, outside retroelements, or immunoglobulins could cause many simultaneous mutations in the genome, a process that can eventually lead to cancer.

## Materials and Methods

We obtained all traces for 10 organisms (600M traces in total), in FASTA format, at the NCBI Trace Archive [48] (http://www.ncbi.nlm.nih.gov/Traces/home/, May 2008) and aligned them with their reference genomes obtained from the UCSC Genome Browser [49]. We did not attempt to filter the initial set of traces by type which would have required the combination of FASTA format sequences with auxiliary information that provides the trace type. Instead we used strict placement criteria, described further below, to obtain the initial dataset summarized in Table 1. We inspected chromatograms for individual traces using the tools provided at the trace archive. We further downloaded SCF raw binary data from the archive, by hand, and analyzed them using Phred version “0.071220.b” [32]. This Phred version can generate an alternate base call for every position in the trace. This results in two sets of sequences for any given trace. By aligning the two sequences from the same trace separately, and looking for a large alignment with a single base-pair offset, we can identify the sequencing error from Figure 1. This might be the basis for an automated test to eliminate this particular sequencing error.

We augmented the above data by downloading auxiliary information and quality scores for a subset of about 20.7 million traces which were, potentially, enriched for editing events. We used runs of three consecutive mismatches of the same type as the enrichment criteria. The number of high quality traces for each editing type (G-to-A, C-to-T, A-to-G, and T-to-C) - is listed in Table 2. For all organisms, except for mosquito and fly, there are more than ten times the number of examples from these four types than the next most frequent type. Furthermore, we extracted the lowest quality subset of these traces enriched for editing to be used for comparison purposes. The number of traces of each editing type from this set, G-to-A, C-to-T, A-to-G, T-to-C, as well as the most frequent or next most frequent type, is listed in Table S5. For Mouse, Human, and *Xenopus tropicalis* these tables also provide (in brackets) the number of traces that likely originated from RNA.

The complete set of mismatches found in these two sets of traces is available to the community as two files, “all.c2.t100.q40+.bed.gz” (5.95MB) and “all.c2.t100.q0-9.bed.gz” (122MB), respectively. The first set is included on the journal's web-site while the second file is available, on request, from the authors. The files contain: the genomic coordinate of the mismatch, the mismatch type, the position on the trace, the quality of the mismatch, the length of the run in which the mismatch was found, the sequencing center, the trace id, the organism, and the likely origin of the trace, DNA or RNA. In order to be counted, each trace must have at least two mismatches with phred 40 or greater that are separated by 100bp or more. Only mismatches with phred scores of 40 or greater are included in the high quality set (see Figures S2, S3, S4 for more data). In the lower quality set, at least two mismatches with phred less than 10 separated by 100bp or more are required. Only mismatches with phred scores of less than 10 are included in the low quality set.

For sequence alignment, we used MegaBlast [50] version 2.2.13 from NCBI. The parameters used were: -W60 (a 60bp seed was selected as a good compromise between computational efficiency and sensitivity, given our requirement of high identity to the reference), -s 400 -p 97 (at least 400bp with 97% identity) -F F (no filtering) -G25 -E10 (these gap and extension penalties preclude insertions and/or deletions in matches). In addition, only unique alignments matching the above criteria were retained. These parameters were chosen for simplicity of subsequent analysis and to reduce the already onerous computational requirements.

Two computational clusters were used to perform the analysis. These clusters were built to assist in deploying data intensive web services [51]. In total, the clusters use a variety of older and newer hardware and consist of 96 nodes w/ (predominantly) 4×1.8GHZ Opteron cores, 4–16GB of RAM per node, and 0–3750GB disk per node. The workflows to generate the initial analysis of the data are written in Perl. The human analysis consumed 347 node days and 530GB of space which was reduced to 22GB of compressed data after parsing the MegaBlast output and discarding redundant matches. A summary of the traces and space/time used by the computation can be found in Table 1. The startling amount of intermediate space required by the mouse analysis, greater than 4.2 terabytes, suggests that many traces in mouse did not place uniquely and consumed large amounts of space, even with our strict chosen cut-offs and using gzip compression on the output of MegaBlast.

## Supporting Information

Dataset S1 Enriched set of editing candidates.
(5.95 MB ZIP)

Dataset S2 Xenopus RNA editing sites.
(0.36 MB TXT)

Figure S1 DNA editing of mouse MMTV-int retrotransposons (both clone mates). DNA editing in a mouse retrotransposon. Two traces (ti#71971190 and ti#71976546 which are mate pairs from one sequencing clone) are aligned to the mouse genomic full length MMTV-int retrotransposon (ERVK family) locus (chr6:68193707-68200951). Both aligned with a large number of G-to-A mismatches, an indication of DNA editing in this active retrotransposon. Additional mismatches are present as well, probably due to the activity of DNA damage proteins.
(0.03 MB DOC)

Figure S2 Substitution spectrum, by quality score, sampled from runs of three substitutions of the same type in ten organisms. In all organisms examined the abundance of G-to-A mismatches dominates all other substitution types for mismatches with Phred quality scores between 10 and 40. From Phred40 and onward the spectrum becomes more even with G-to-A, C-to-T, A-to-G and T-to-C all roughly the same with each of those mismatch types representing 20% of all substitutions.
(0.06 MB TIF)

Figure S3 Absolute abundance of mismatches in human w/100 bp runs. Shows absolute abundance of runs from Figure 1A.
(0.03 MB TIF)

Figure S4 Absolute abundance of mismatches in human. Shows absolute abundance of runs from Figure 1A, removing the 100 bp restriction.
(0.08 MB TIF)

Table S1 Summary of traces without enrichment (RNA origin) by mismatch type. “Other” indicates the most abundant type other than those listed. No enrichment for the ADAR derived mismatches are observed in the full set.
(0.03 MB DOC)

Table S2 Sequence context preceding mismatch (enriched, higher quality, RNA). There is a clear under representation of the “G” nucleotide upstream to the mismatch, in agreement with known ADAR signatures in both human and Xenopus. RNA editing is known to be less common in mouse, thus, this is consistent with a lack of depletion.
(0.03 MB DOC)

Table S3 Sequence context preceding mismatch (not enriched, RNA). The position preceding an edited site is known to be depleted in “g”. We looked at the position preceding an A-to-G or T-to-C mismatch in RNA derived traces. The depletion is clearly visible in the enriched set (see Materials and Methods) but no such signature was observed in the complete set of RNA derived traces.
(0.03 MB DOC)

Table S4 Summary of Traces without enrichment (RNA origin). “unique bp” indicates the total number of genomic positions covered by the placed traces of the RNA traces.
(0.03 MB DOC)

Table S5 Editing enriched traces-lower quality. Number of traces, by mismatch type, with two or more mismatch below a quality threshold of Phred 10, spanning 100 bp or more. For Mouse, Human, and *Xenopus tropicalis* these tables also provide (in brackets) the number of traces that likely originated from RNA. See Materials and Methods for more details.
(0.03 MB DOC)
