# Supplementary material for: A Survey of Genomic Traces Reveals a Common Sequencing Error, RNA Editing, and DNA Editing
Source: PLoS Genet. 2010 May 20;6(5):e1000954. doi: 10.1371/journal.pgen.1000954 (PMC2873906; doi:10.1371/journal.pgen.1000954)
Supplement: Figure S1 — DNA editing of mouse MMTV-int retrotransposons (both clone mates). DNA editing in a mouse retrotransposon. Two traces (ti#71971190 and ti#71976546 which are mate pairs from one sequencing clone) are aligned to the mouse genomic full length MMTV-int retrotransposon (ERVK family) locus (chr6:68193707-68200951). Both aligned with a large number of G-to-A mismatches, an indication of DNA editing in this active retrotransposon. Additional mismatches are present as well, probably due to the activity of DNA damage proteins. (0.03 MB DOC) [file pgen.1000954.s003.doc]

Trace id: 71971190

Genome 1 AAAGATCTTCT-CACAGGGTCC-TGGAAAGGACCCG-ATGTCCTAATAACAGCCGGACGA 57

**Trace**  958 ...........**T**..........**C**..**A**..........**G**....................... 899

Genome 58 GGCTATGCTTGTGTTTTTCCACAGGATGCCGAATCACCAATTTGGGTCCCTGACCGATT- 116

**Trace**  898 ..............................**A**.......**C**.................**G**..**T** 839

Genome 117 CATCCGACCTTTCACTGAACGGAAGGGATCGACGCCCACGCCTAGCGCTGCGGAGAAAAC 176

**Trace**  838 ..................**G**......................................... 779

Genome 177 GCCGCCGCGAGATGAGAAAGATCACCAAGAAAGTCCGGAAAATGACCCTAGACCCCATCA 236

**Trace**  778 ...............**A**............................................ 719

Genome 237 AAGAAAAGACGGCTTGGCAACAACTGCAGGCGTTGATCTCCGAAGCGGAGGAGGTTCTTA 296

Trace 718 ............................................................ 659

Genome 297 AAACCTCACAAACTCCCCAAACCTCTCTGACCTTATTTCTTGCTTTGTTGTCTGTCCTCG 356

**Trace**  658 ..........................**T**................................. 599

Genome 357 GCCCCCTGCCTGTGACCGGGGAAAGTTATTGGGCTTACCTACCTAAACCACCTATTCTCC 416

**Trace**  598 ....................**A**....................................... 539

Genome 417 ATCCCGTGGGATGGGGAAGTACAGACCCCATTAGAGTTCTGACAAATCAAACCATGTATT 476

**Trace**  538 .......**A**......**AA**.................**A**.**A**........................ 479

Genome 477 TGGGTGGTTCGCCTGACTTTCACGGGTTTAGAAACATGTCTGGTAATGTACATTTTGAGG 536

Trace 478 ............................................................ 419

Genome 537 GGAAGTCTGATACGCTCCCCATTTGCTTTTCCTTCTCCTTTTCTACCCCCACGGGCTGCT 596

Trace 418 ............................................................ 359

Genome 597 TTCAAGTAGATAAGCAAGTATTTCTTTCTGATACACCCACGGTTGATAATAATAAACCTG 656

**Trace**  358 ................**G**........................................... 299

Genome 657 GGGGAAAGGGTGATAAAAGGCGTATGTGGGAACTTTGGTTGACTACCTTGGGGAACTCGG 716

**Trace**  298 ..**AA**.......................................**C**..**T**..**A**.**AA**.....**A**. 239

Genome 717 GGGCCAATACAAAACTGGTCCCTATAAAAAAGAAGTTGCCCCCCAAATATCCTCACTGCC 776

Trace 238 ............................................................ 179

Genome 777 AGATCGCCTTTAAGAAGGACGCCTTCTGGGAGGGAGACGAGTCTGCTCCTCCACGGTGGT 836

**Trace**  178 .**A**.......**C**...**A**.............................................. 119

Genome 837 TGCCTTGCGCCTTCCCTGACCAGGGGGTGAGTTTTTCTCCAAAAGGGGCCCTTGGGTTAC 896

**Trace**  118 .....................................................**AA**..... 59

Genome 897 TTTGGGATTTCTCCCTTCCCTCGCCTAG 924

**Trace**  58 ....**A**....................... 31

Alignment of the matepair clone:

Score = 1390 bits (723), Expect = 0.0

Identities = 858/908 (94%), Gaps = 7/908 (0%)

Strand=Plus/Plus

Genome 7 GGATTGATAATAGGTAGGAGTTCCAATTATAAAAAGGGACTTGAGGTTTTACCAGGGGTC 66

**Trace**  43 .....**A**..........**AA**.......................**C**..............**A**... 102

Genome 67 ATTGACTCCGATTTCCAAGGAGAAATCAAGGTTATGGTTAAGGCCGCAAAAAATGCGGTC 126

Trace 103 ............................................................ 162

Genome 127 ATCATTCACAAAGGAGAAAGAATAGCACAACTACTGTTGCTGCCATATTTAAAATTGCCC 186

**Trace**  163 ...................**A**........................................ 222

Genome 187 AATCCTATAATCAAGGAAGAACGAGGCTCAGAAGGCTTCGGATCAACAAGTCATGTACAT 246

**Trace**  223 ........**T**.....**A**.................**G**........................... 282

Genome 247 TGGGTGCAGGAAATAAGTGATTCTAGACCCATGCTTCATATTTACTTGAATGGAAGAAGA 306

**Trace**  283 ........**A**......................................**A**....**A**..**A**.... 342

Genome 307 TTCCTCGGTCTCTTAGATACCGGGGCAGATAAAACTTGCATAGCAGGTAAGGACTGGCCA 366

Trace 343 ............................................................ 402

Genome 367 GCTAATTGGCCTATTCACCAAACTGAAAATTCTCTCCAAGGTTTAGGCATGGCCTGTGGG 426

**Trace**  403 ...........**C**............**A**................................... 462

Genome 427 GTGGCACGTAGTAGTCAGCCACTCCGCTGGCAACATGAGGATAAATCAGGGATTATACAT 486

**Trace**  463 ......................................**A**..........**A**.........**C** 522

Genome 487 CCCTTTGTGATCCCTACACTGCCCTTTACCTTGTGGGGAAGAGACATTATGAAAGAGATA 546

**Trace**  523 ..**T**...............**T**....**T**..**C**.....**A**...........**T**............... 582

Genome 547 AAGGTCAGGTTAATGACTGACTCACCAGATGATTCACAGGATTTATGATAGGGGCCATTG 606

**Trace**  583 ........**A**........**A**....................**A**..................... 642

Genome 607 AGAGCAATCTCTTTGCAGACCAAATATCTTGGAAATCAGACCAGCCTGTATGGCTTAATC 666

**Trace**  643 ........................................**T**................... 702

Genome 667 AATGGCCCCTTAGACAAGAAAAGTTACA-GGCTTTACAACAGTTAGTGACAGAACAATTA 725

**Trace**  703 ............**A**...............**G**............................... 762

Genome 726 CAACTGGGCCACTTAGAAGAGAGCAATAGCCCTTGGAATACGCCT-GTTTTTGTCATTAA 784

**Trace**  763 ...**T**...........**A**..................**AA**.........**G**.............. 822

Genome 785 AAAGAAGTCAGGAAAAT-GGAGGCTGTTGCAAGACCTACGTGCAGTTAATGCCACAATGC 843

**Trace**  823 ...**A**.............**G**........**C**.**A**.**-**............................. 881

Genome 844 ACGATAT-GGGAGCATTA-CAACCAGGCTTG-CCGTCCCCTGTAGCAGTCCCTAAAGGAT 900

**Trace**  882 .......**G**....**A**.....**C**............**C**........**CTGTA**...........**A**... 941

Genome 901 GGGAAATA 908

Trace 942 ........ 949
