# Supplementary figures and images for: A Survey of Genomic Traces Reveals a Common Sequencing Error, RNA Editing, and DNA Editing
Source: PLoS Genet. 2010 May 20;6(5):e1000954. doi: 10.1371/journal.pgen.1000954 (PMC2873906; doi:10.1371/journal.pgen.1000954)

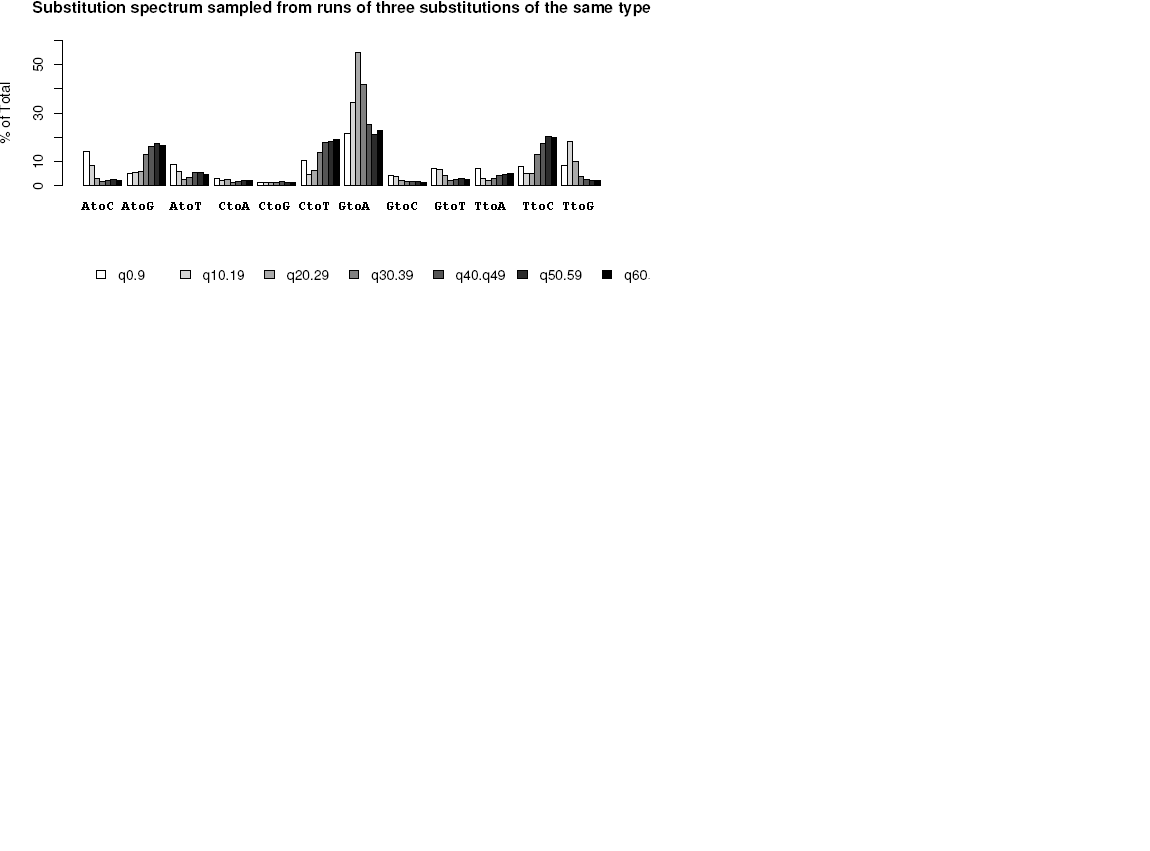

Supplement: Figure S2 — Substitution spectrum, by quality score, sampled from runs of three substitutions of the same type in ten organisms. In all organisms examined the abundance of G-to-A mismatches dominates all other substitution types for mismatches with Phred quality scores between 10 and 40. From Phred40 and onward the spectrum becomes more even with G-to-A, C-to-T, A-to-G and T-to-C all roughly the same with each of those mismatch types representing 20% of all substitutions. (0.06 MB TIF) [file pgen.1000954.s004.tif]

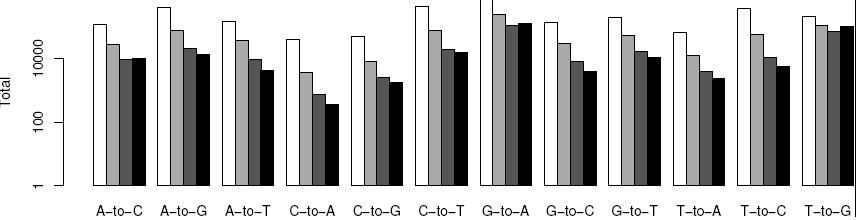

Supplement: Figure S3 — Absolute abundance of mismatches in human w/100 bp runs. Shows absolute abundance of runs from Figure 1A. (0.03 MB TIF) [file pgen.1000954.s005.tif]

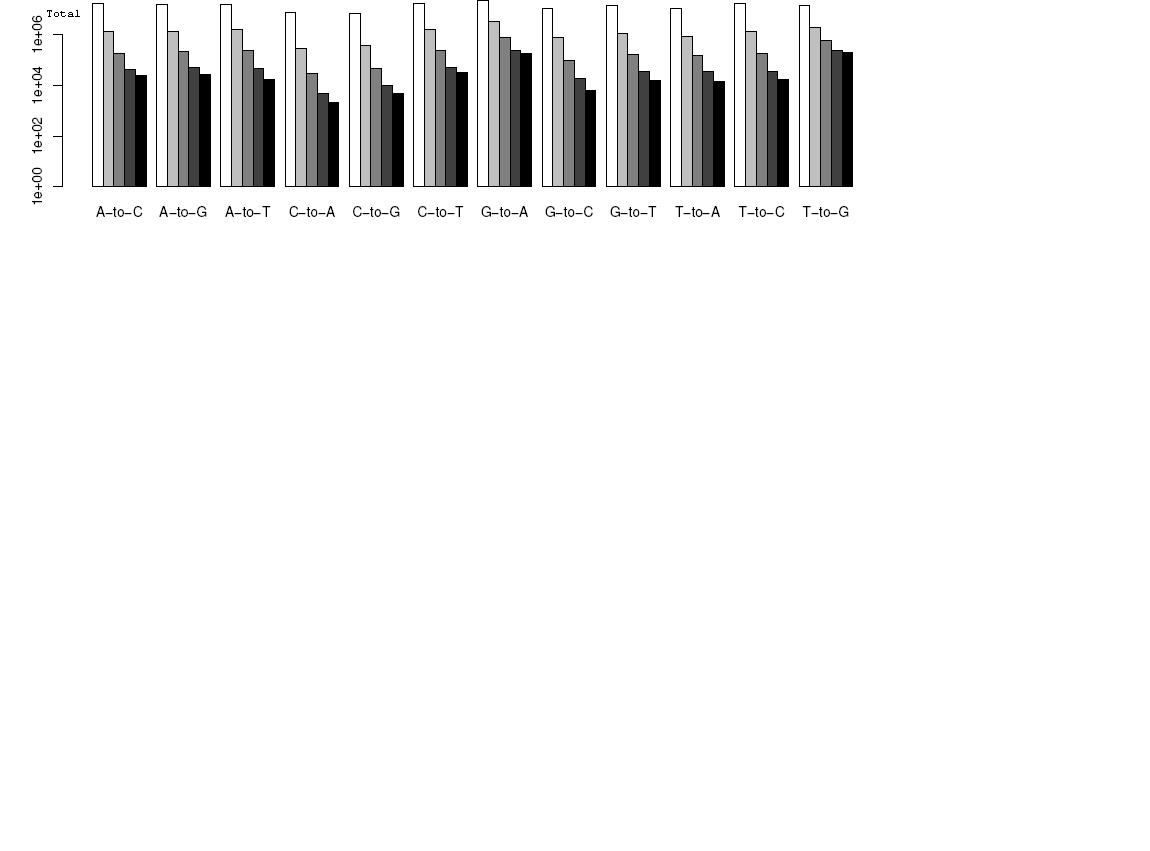

Supplement: Figure S4 — Absolute abundance of mismatches in human. Shows absolute abundance of runs from Figure 1A, removing the 100 bp restriction. (0.08 MB TIF) [file pgen.1000954.s006.tif]
