# Supplementary material for: A Survey of Genomic Traces Reveals a Common Sequencing Error, RNA Editing, and DNA Editing
Source: PLoS Genet. 2010 May 20;6(5):e1000954. doi: 10.1371/journal.pgen.1000954 (PMC2873906; doi:10.1371/journal.pgen.1000954)
Supplement: Table S1 — Summary of traces without enrichment (RNA origin) by mismatch type. “Other” indicates the most abundant type other than those listed. No enrichment for the ADAR derived mismatches are observed in the full set. (0.03 MB DOC) [file pgen.1000954.s007.doc]

### Table S1. Summary of traces without enrichment (RNA origin) by mismatch type:

“Other” indicates the most abundant type other than those listed. No enrichment for the ADAR derived mismatches are observed in the full set.

| reference Genome | G-to-A | C-to-T | A-to-G | T-to-C | Other |
| --- | --- | --- | --- | --- | --- |
| hg18 | 63,850 | 44,896 | 45,749 | 44,298 | 39,577 |
| mm9 | 163,695 | 151,057 | 114,538 | 148,471 | 153,104 |
| xenTro2 | 106,270 | 77,682 | 83,143 | 88,415 | 89,016 |
|  |  |  |  |  |  |
