# Supplementary material for: A Survey of Genomic Traces Reveals a Common Sequencing Error, RNA Editing, and DNA Editing
Source: PLoS Genet. 2010 May 20;6(5):e1000954. doi: 10.1371/journal.pgen.1000954 (PMC2873906; doi:10.1371/journal.pgen.1000954)
Supplement: Table S2 — Sequence context preceding mismatch (enriched, higher quality, RNA). There is a clear under representation of the “G” nucleotide upstream to the mismatch, in agreement with known ADAR signatures in both human and Xenopus. RNA editing is known to be less common in mouse, thus, this is consistent with a lack of depletion. (0.03 MB DOC) [file pgen.1000954.s008.doc]

### Table S2. Sequence context preceding mismatch (enriched, higher quality, RNA):

There is a clear under representation of the “G” nucleotide upstream to the mismatch, in agreement with known ADAR signatures in both human and Xenopus. RNA editing is known to be less common in mouse, thus, this is consistent with a lack of depletion.

| **Genome** | a | c | g | t | total |
| --- | --- | --- | --- | --- | --- |
| Human | 1117 | 1126 | 265 (7.8%) | 866 | 3374 |
| Mouse | 452 | 624 | 394 (20.7%) | 426 | 1896 |
| Xenopus | 6586 | 3221 | 1062 (5.8%) | 7292 | 18161 |
|  |  |  |  |  |  |
