# Supplementary material for: A Survey of Genomic Traces Reveals a Common Sequencing Error, RNA Editing, and DNA Editing
Source: PLoS Genet. 2010 May 20;6(5):e1000954. doi: 10.1371/journal.pgen.1000954 (PMC2873906; doi:10.1371/journal.pgen.1000954)
Supplement: Table S3 — Sequence context preceding mismatch (not enriched, RNA). The position preceding an edited site is known to be depleted in “g”. We looked at the position preceding an A-to-G or T-to-C mismatch in RNA derived traces. The depletion is clearly visible in the enriched set (see Materials and Methods) but no such signature was observed in the complete set of RNA derived traces. (0.03 MB DOC) [file pgen.1000954.s009.doc]

### Table S3. Sequence context preceding mismatch (not enriched, RNA)

The position preceding an edited site is known to be depleted in “g”. We looked at the position preceding an A-to-G or T-to-C mismatch in RNA derived traces. The depletion is clearly visible in the enriched set (see Methods) but no such signature was observed in the complete set of RNA derived traces.

| **Genome** | a | c | g | t | total |
| --- | --- | --- | --- | --- | --- |
| Human | 36,694 | 36,894 | 41,661 (30.3%) | 22,064 | 137,313 |
| Mouse | 139,019 | 95,437 | 103,807 (25.5%) | 69,436 | 407,699 |
| Xenopus | 78,246 | 61,714 | 62,677 (23.6%) | 62,387 | 265,024 |
|  |  |  |  |  |  |
