# Supplementary material for: A Survey of Genomic Traces Reveals a Common Sequencing Error, RNA Editing, and DNA Editing
Source: PLoS Genet. 2010 May 20;6(5):e1000954. doi: 10.1371/journal.pgen.1000954 (PMC2873906; doi:10.1371/journal.pgen.1000954)
Supplement: Table S4 — Summary of Traces without enrichment (RNA origin). “unique bp” indicates the total number of genomic positions covered by the placed traces of the RNA traces. (0.03 MB DOC) [file pgen.1000954.s010.doc]

### Table S4. Summary of Traces without enrichment (RNA origin):

“unique bp” indicates the total number of genomic positions covered by the placed traces of the RNA traces.

| filename | placed traces | placed bp | unique bp | avg. trace length (bp) | total traces |
| --- | --- | --- | --- | --- | --- |
| hg18.0369.rna.bed.gz | 252,946 | 150,317,259 | 38,550,612 | 594 | 720,462 |
| mm9.6f91.rna.bed.gz | 512,602 | 326,506,519 | 183,249,088 | 637 | 1,376,201 |
| xenTro2.0b48.rna.bed.gz | 454,183 | 299,833,478 | 55,979,419 | 660 | 1,253,617 |
