# Supplementary material for: A Survey of Genomic Traces Reveals a Common Sequencing Error, RNA Editing, and DNA Editing
Source: PLoS Genet. 2010 May 20;6(5):e1000954. doi: 10.1371/journal.pgen.1000954 (PMC2873906; doi:10.1371/journal.pgen.1000954)
Supplement: Table S5 — Editing enriched traces-lower quality. Number of traces, by mismatch type, with two or more mismatch below a quality threshold of Phred 10, spanning 100 bp or more. For Mouse, Human, and Xenopus tropicalis these tables also provide (in brackets) the number of traces that likely originated from RNA. See Materials and Methods for more details. (0.03 MB DOC) [file pgen.1000954.s011.doc]

### Table S5. Editing enriched traces—lower quality:

Number of traces, by mismatch type, with two or more mismatch below a quality threshold of Phred 10, spanning 100bp or more. For Mouse, Human, and *Xenopus tropicalis* these tables also provide (in brackets) the number of traces that likely originated from RNA. See Methods for more details.

| **Reference genome version** | **G-to-A** | **C-to-T** | **A-to-G** | **T-to-C** | **Other** |
| --- | --- | --- | --- | --- | --- |
| anoGam1 | 980 | 190 | 3265 | 659 | 9647 |
| calJac1 | 27445 | 15166 | 17039 | 1064 | 17765 |
| canFam2 | 4654 | 107275 | 9652 | 4803 | 31217 |
| dm3 | 1448 | 222 | 166 | 535 | 4982 |
| galGal3 | 48635 | 6691 | 2708 | 4064 | 9418 |
| hg18 | 108190(174) | 39595(111) | 21880(120) | 11805(67) | 64374(1379) |
| mm9 | 343435(1147) | 116745(330) | 75907(354) | 67740(231) | 141660(739) |
| panTro2 | 118843 | 44310 | 6839 | 8143 | 34006 |
| fr2 | 369 | 214 | 121 | 872 | 3390 |
| xenTro2 | 1451(181) | 1189(7) | 963(54) | 13678(27) | 41160(157) |
